# Hierarchical graphs for rule-based modeling of biochemical systems

**DOI:** 10.1186/1471-2105-12-45

**Published:** 2011-02-02

**Authors:** Nathan W Lemons, Bin Hu, William S Hlavacek

**Affiliations:** 1 Department of Mathematics and its Applications, Central European University, H-1051 Budapest, Hungary; 2 Theoretical Biology and Biophysics Group, Theoretical Division, Los Alamos National Laboratory, Los Alamos, NM 87545, USA; 3 Center for Nonlinear Studies, Los Alamos National Laboratory, Los Alamos, NM 87545, USA; 4 Department of Biology, University of New Mexico, Albuquerque, NM 87131, USA

## Abstract

**Background:**

In rule-based modeling, graphs are used to represent molecules: a colored vertex represents a component of a molecule, a vertex attribute represents the internal state of a component, and an edge represents a bond between components. Components of a molecule share the same color. Furthermore, graph-rewriting rules are used to represent molecular interactions. A rule that specifies addition (removal) of an edge represents a class of association (dissociation) reactions, and a rule that specifies a change of a vertex attribute represents a class of reactions that affect the internal state of a molecular component. A set of rules comprises an executable model that can be used to determine, through various means, the system-level dynamics of molecular interactions in a biochemical system.

**Results:**

For purposes of model annotation, we propose the use of hierarchical graphs to represent structural relationships among components and subcomponents of molecules. We illustrate how hierarchical graphs can be used to naturally document the structural organization of the functional components and subcomponents of two proteins: the protein tyrosine kinase Lck and the T cell receptor (TCR) complex. We also show that computational methods developed for regular graphs can be applied to hierarchical graphs. In particular, we describe a generalization of Nauty, a graph isomorphism and canonical labeling algorithm. The generalized version of the Nauty procedure, which we call HNauty, can be used to assign canonical labels to hierarchical graphs or more generally to graphs with multiple edge types. The difference between the Nauty and HNauty procedures is minor, but for completeness, we provide an explanation of the entire HNauty algorithm.

**Conclusions:**

Hierarchical graphs provide more intuitive formal representations of proteins and other structured molecules with multiple functional components than do the regular graphs of current languages for specifying rule-based models, such as the BioNetGen language (BNGL). Thus, the proposed use of hierarchical graphs should promote clarity and better understanding of rule-based models.

## Background

Our predictive understanding of cell signaling is limited, in part because it is difficult to fully capture in a conventional model, such as a system of coupled ordinary differential equations (ODEs), the system-level dynamics of molecular interactions that mediate cell signaling [1-3]. A major reason is combinatorial complexity [4-6], the potential for molecular interactions to generate a large number of chemically distinguishable molecular states and molecular complexes. One cause of combinatorial complexity is multisite phosphorylation [7-9]. Another is multivalent binding, which can mediate polymerization-like reactions that produce a distribution of oligomers [10,11]. Combinatorial complexity is an inherent feature of cell signaling, because a typical signaling protein contains multiple functional components [12]. These components can include a protein interaction domain, such as a Src homology 2 (SH2) or SH3 domain [13-15]; a catalytic domain, such as a protein tyrosine kinase (PTK) [16,17]; a linear motif [18], such as a proline-rich sequence (PRS) recognized by SH3 domains or an immunoreceptor tyrosine-based activation or inhibition motif (ITAM or ITIM) [19,20]; and one or more sites of post-translational modification, with a multitude of modifications being possible [21]. Prominent examples of post-translational modifications include serine, threonine and tyrosine (S/T/Y) phosphorylation, which is governed by antagonistic activities of kinases and phosphatases [22,23], and ubiquitination, which is mediated by E3 ubiquitin ligases and other proteins [24,25].

Combinatorial complexity limits the application of conventional modeling approaches such as ODEs, because specification of a conventional model requires that one be able to list the possible reactions in a system, or the equivalent [3]. To overcome this problem, a new modeling approach has been developed: rule-based modeling [3,26]. In this approach, a model is specified in terms of rules for molecular interactions, rather than in terms of a list of possible reactions. Reactions are implied by rules, and these reactions can be found in principle and sometimes in practice [27-29], but there is no need to enumerate the possible reactions in a system to formulate or simulate a model [30-32]. A variety of algorithms and software tools have been developed for simulating rule-based models, including tools that account for steric effects and diffusion [27-45], and these tools have been applied to study various aspects of a number of cell signaling systems [34,46-50]. It is now possible to formulate and simulate models that account comprehensively for the large numbers of molecules and molecular interactions that typically comprise a cell signaling system, which raises the issue of how to annotate and visualize large-scale rule-based models.

Visualization of the elements of a rule-based model is natural to some extent because rule-based modeling, at least in some realizations, is based on or can be interpreted as being based on an underlying graphical formalism [51], which serves as the foundation for the BioNetGen language (BNGL) [29]. This model-specification language is supported by anumber of softwaretools [27,29,32,40,44,45]. Another model-specification languageisKappa [26,41,52,53], which is closely related to BNGL. In the BNGL formalism, which is briefly summarized in this section and described in greater detail below, graphs are used to represent molecules, and graph-rewriting rules are used to represent molecular interactions.

In a rule-based model for a cell signaling system, the graphs of a model typically represent proteins, which are taken to be the building blocks of most chemical species in the system. These graphs can be visualized according to the conventions of Faeder et al. [54]. A graph representing a protein includes a colored vertex for each functional component of the protein. The color represents the type of protein being represented by a graph, i.e., the protein name is essentially the color of the graph representing the protein. The vertices of graphs can be associated with variable attributes to represent so-called internal states of components. An internal state is an abstraction that is often useful for representing, for example, the phosphorylation status of an amino acid residue. In graphs for molecular complexes, edges are used to represent bonds between molecular components. Thus, the composition and the connectivity of a molecular complex (but not usually its three-dimensional structure) are tracked explicitly in a BNGL-encoded rule-based model.

In general, the graph-rewriting rules in a BNGL-encoded model specify simple operations on graphs, which define the outcomes of molecular interactions: the addition of an edge to represent an association event, the removal of an edge to represent a dissociation event, or the change of a vertex attribute to represent an internal state change, such as a post-translational modification event. Rules can also be specified for synthesis and degradation reactions [29]. Two important features of a rule are the specification of a reaction center (the set of components directly affected by a molecular interaction) and the specification of the molecular context in which a molecular interaction occurs, i.e., the necessary and sufficient conditions that must be satisfied for a reaction to occur. Another feature of a rule is an associated rate law, which is used to characterize all reactions implied by the rule up to statistical factors, which are derived from the properties of reactants [28,51]. Thus, a rule can be interpreted as providing a coarse-grained definition of a class of reactions that arises from a particular interaction, with each reaction implied by a rule involving a common transformation and rate law. The granularity of a rule is adjustable.

Although the rule-based modeling framework described above is expressive and sufficiently rich to describe a wide array of molecular interactions involved in cell signaling, the graphs of this framework are not sufficiently expressive to provide a completely natural representation of the substructures of signaling proteins. As discussed in detail below, components of a protein can themselves contain components, and so on. Yet, in the framework described above, the components of a protein, regardless of their structural relationships, are represented in the same way, as the colored vertices of a graph, with a shared color indicating joint membership in the set of components of a particular type of molecule. In other words, if a component and a subcomponent of this component are both included in a model, the structural relationship between the component and subcomponent is lost. This representational limitation may not prevent a modeler from specifying a model with desired properties, but it may prevent others from easily connecting the formal elements of the model to the underlying biology and easily interpreting the model as intended.

Here, mainly to enable better annotation of rule-based models, we introduce the concept of using hierarchical graphs to represent molecules, such as proteins, for which there are structural relationships among component parts. We also present an algorithm and software, which we have called HNauty, for assigning canonical labels to hierarchical graphs. Canonical labeling enables one to determine if two graphs are the same or different simply by comparing their labels. This task, which is essentially equivalent to the solution of a graph isomorphism problem, is a routine part of network generation, the process of enumerating the reactions implied by a set of rules. Network generation, which is not always practical, is an essential ingredient in the generate-first and on-the-fly approaches to simulation of a rule-based model [27,28,51]. Thus, this report not only lays groundwork for using hierarchical graphs to annotate rule-based models but also lays groundwork for making such graphs elements of executable models.

In the remainder of this section, we provide additional background on the graphical formalism underlying BNGL, on the hierarchical substructures of proteins, and on graph isomorphism and Nauty [55], a software tool for canonical labeling of colored graphs. We then provide examples of how hierarchical graphs can be used to represent proteins more naturally than the graphs of the BNGL formalism, and we present a simple extension of the method implemented in Nauty that allows for canonical labeling of hierarchical graphs. Finally, we present and evaluate our implementation of the extended canonical labeling tool, HNauty. The HNauty software is freely available; the source code is provided as Additional files 1 and 2. Its functionality can also be accessed from within BioNetGen [29].

## Methods

### Graphical formalism underlying the BioNetGen Language

The model-specification language BNGL has evolved over time and has been described in detail [3,27-29]. It is based on a graphical formalism described initially by Faeder et al. [28,54] and then more formally and in greater detail by Blinov et al. [51]. The formalism includes various types of graphs, two of which are relevant for our purposes: the molecular entity graph and the chemical species graph. Let us recall the basic definition of a graph. A graph is a pair (V,ℰ) where V is a finite set (called the vertex set) and ℰ is a collection of pairs of vertices. A simple graph is a graph in which there is at most one edge between any two vertices. If this condition does not hold and the graph has multiple edges between at least one pair of vertices, then the graph is a multi-graph. All graphs are assumed to be simple unless otherwise noted. If a graph is directed, then the edges are ordered pairs; otherwise they are unordered. A labeled graph is a graph G=(V,ℰ) together with a set of labels, *L *and a map $l_{V}:V\to L$. It is also possible to label the edges via a map $l_{ℰ}:ℰ\to L$. In an attributed graph, some vertices, in addition to fixed labels, are associated with variable attributes, which are used in BNGL to represent internal states of components.

We are now ready to introduce the two types of graphs in the BNGL formalism that are of interest here. A molecular entity graph is a labeled graph together with a map that assigns to each vertex a list of possible attributes. A chemical species graph is derived from a molecular entity graph or a collection of connected molecular entity graphs such that all variable attributes take on specific values. Thus, molecular entity graphs model the types of molecules in a reaction network and chemical species graphs model specific chemical species, which are composed of molecules. These two types of graphs can be encoded in a machine-readable form according to the conventions of BNGL [29]. As should be apparent from the above definitions, in models specified using BNGL, all components (vertices) of proteins (graphs) are considered structurally equivalent (i.e., there are no subcomponents within components). Thus, the graphs of BNGL can potentially obscure the structural relationships among the component parts of a protein.

### Two examples of proteins with hierarchical substructures

**Figure 1:**
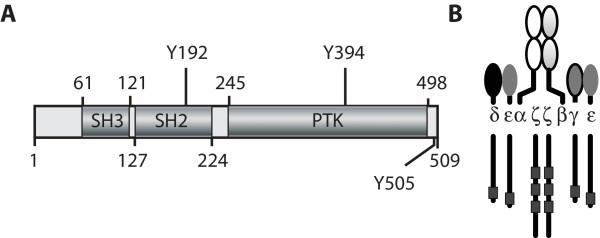
**Lck and TCR complex**. a) The hierarchical substructure of protein tyrosine kinase Lck. There are three domains in Lck: a Src homology 3 (SH3) domain, a Src homology 2 (SH2) domain, and a protein tyrosine kinase (PTK) domain. Three tyrosine residues are subject to phosphorylation and dephosphorylation (Y192, Y394 and Y505). b) The structure of the TCR complex. The TCR complex is composed of *αβ*, γϵ, and *δϵ *heterodimers and a *ζζ *homodimer. Black boxes represent ITAMs.

Here, we discuss two examples of proteins with hierarchical substructures (Figure 1), meaning that functional components in these proteins have subcomponents.

Figure 1A depicts the human lymphocyte cell-specific protein-tyrosine kinase (Lck), which is a Src-family non-receptor tyrosine kinase that plays an important role in T cell receptor (TCR) signaling [56,57]. As can be seen, Lck is composed of one SH3 domain, one SH2 domain, and one PTK domain [58]. The tyrosine residues of Lck represented in Figure 1A (Y192, Y394 and Y505) have been shown to be phosphorylated during TCR signaling [59]. Phosphorylation of Y192 in the SH2 domain of Lck reduces the ability of the SH2 domain to bind its phosphotyrosine-containing binding sites in other proteins [60]. Autophosphorylation of Y394, which is located in the activation loop of the PTK domain of Lck, increases the kinase activity of Lck [61]. Phosphorylation of Y505 allows an intramolecular interaction between pY505 and the SH2 domain of Lck, which downregulates Lck kinase activity through the resulting conformational change of the protein [62]. A depiction of Lck and its above mentioned components in the graphical formalism of BNGL would only show that there are three domains and three tyrosine residues in Lck. There would be no indication that Y192 is part of the SH2 domain or that Y394 is part of the PTK domain. Below, we will show that these relationships are clear from a hierarchical graph representation of Lck. The hierarchical graphs that will be formally introduced later include directed edges to indicate structural relationships. An edge directed from a component to a subcomponent can be interpreted to mean that the subcomponent is part of the component.

Figure 1B depicts the TCR complex, a multimeric protein expressed on the surface of T lymphocytes. The TCR complex has a subunit responsible for recognition of peptide antigens, which is composed of disulfide-linked α and β chains. It also has a number of subunits responsible for interacting with cytoplasmic signaling proteins. Two subunits are composed of the CD3γ, *δ *and ϵ chains, which each contain an ITAM and which form two disulfide-linked heterodimers: a γϵ heterodimer and a *δ*ϵ heterodimer. Finally, there is a homodimer of disulfide-linked ζ chains, which each contain three ITAMs. Each ITAM in the TCR complex contains two tyrosine residues, which are dynamically phosphorylated and dephosphorylated during TCR signaling. A tyrosine residue in the ITAM of CD3ϵ, Y188, is also part of a PRS that contains the motif PxxDY. It is important to recognize the structural overlap between the PRS and ITAM of CD3ϵ, because phosphorylation of Y188 inhibits interaction of the Y188-containing PRS with SH3 domains and SH3 domain binding at the PRS inhibits phosphorylation of Y188 [63]. The structural relationships discussed above cannot be explicitly represented using the regular graphs of BNGL. Below, we will show that these relationships are clear from a hierarchical graph representation of the TCR complex.

### Graph isomorphism

Graphs that are essentially the same are called isomorphic (the exact definition is given below). As described elsewhere [29], to generate a reaction network from a set of rules, BioNetGen [54] must determine, upon generation of a chemical species graph, if the graph has already been generated, i.e., if it is already part of the reaction network. If the graph does not already exist in the network, it is added to the reaction network. Specifically, upon generation of a chemical species graph, the newly generated graph must be checked for isomorphism with every other existing chemical species graph in the reaction network. To reduce the time necessary for this procedure, BioNetGen assigns to each chemical species graph a canonical label (which is the same for isomorphic graphs), or for computational efficiency, a pseudo canonical label, which is not guaranteed to be unique but often is in practice. Here, we will only be concerned with true canonical labels, but in the case of either a canonical or pseudo canonical labeling algorithm, the algorithm must be called only once for each chemical species graph representing a newly generated reaction product. An algorithm assigning canonical labels can thus be used to determine graph isomorphism efficiently, as string comparisons are much more efficient than graph comparisons. In practice, if there are a large number of graphs that need to be compared to one another, it is efficient to assign canonical labels using an algorithm such as Nauty [55,64] to each graph and then to compare the graphs using their labels.

Although hierarchical graphs are currently only proposed here for annotation purposes, such graphs could in principle be incorporated into models as formal elements. To enable the incorporation of hierarchical graphs into executable models, we describe a generalization of the Nauty algorithm [55], which takes as input hierarchical graphs and assigns them canonical labels.

## Results

### Hierarchical Graphs for Annotating Rule-based Models

#### Definitions

We give exact definitions of hierarchical graphs before discussing how hierarchical graphs can be used to represent particular proteins with hierarchical substructures.

A hierarchical graph is a graph G=(V,ℰ) together with an acyclic parent function p:V→V. The parent function defines the hierarchy; the parent of a vertex is the next level up in the hierarchy. While the function p must be acyclic we do allow vertices to be their own parents; the assignment *p*(*v*) = *v *is permissible (and indeed such a relationship must hold for at least one vertex in the graph). It is common to represent the hierarchy as a directed tree (or forest). A labeled hierarchical graph is a hierarchical graph with a labeling of the vertices (and or edges) as above. Although many proteins do indeed have a hierarchical substructure, the above definition may be too strict in some cases. An example of such a case is provided by overlapping linear motifs (e.g., the overlapping PRS and ITAM in CD3ϵ), because amino acid residues in the region of overlap cannot be considered to have a unique parent in a hierarchical graph. We will call such hierarchies pseudo-hierarchies and define a pseudo-hierarchical graph to be a directed acyclic graph. Although individual nodes in pseudo-hierarchical graphs may not have a unique parent, the acyclicity of the hierarchy ensures there is still a "top-down" structure to the graph.

In models, we will want to essentially use both hierarchical (or pseudo-hierarchical) graphs and the conventional flat graphs of BNGL at the same time: the first type of graph to show the structural relationships between molecular components and the second type of graph to show bonds between molecular components. Thus, we will use graphs with two edge types, the first type will represent the (pseudo-)hierarchy and will be directed, the second type will represent bonds and will be undirected.

The vertices of the graphs in BNGL are not only labeled but are also attributed. This means, for example, that a vertex that represents a site of phosphorylation on a protein may either have the attribute "phosphorylated" or the attribute "not phosphorylated." Technically, the label of a vertex stays the same, whereas an attribute of a vertex can change. This concept of variable attributes or internal states reflects an understanding that a protein is essentially the same molecule whether or not one of its amino acid residues is phosphorylated. Formally, each vertex is assigned a list of possible attributes and then each vertex is assigned an attribute from the corresponding list. In BNGL, labels cannot change during a simulation of a model; attributes can. Hierarchical graphs can be attributed in the same manner.

#### Hierarchical graph representation of Lck

**Figure 2:**
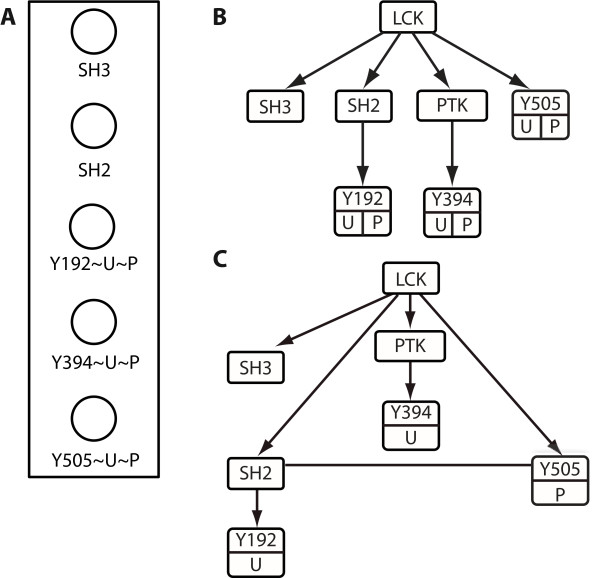
**Graphical representations of Lck**. a) Lck is represented by a molecular entity graph [27,67]. Each component in a protein is represented by a circle. The box that contains all these circles represents the Lck molecule. b) Domains and sites of post-translational modification in Lck are represented in a hierarchical molecular entity graph. Arrows indicate the containing relationships. Each component is represented as a node in the graph. The tyrosine residues Y192, Y394, and Y505 have phosphorylated (P) and unphosphorylated (U) internal states. This graph follows the conventions used in GetBonNie [40]. c) Lck is represented by a hierarchical chemical species graph. In a chemical species graph, the state of each domain or site that has more than one internal state is clearly defined. Here, "U" and "P" under Y192 and Y505 specify that Y192 and Y505 can be in unphosphorylated and phosphorylated states, respectively. The undirected line (with no arrow) between SH2 and Y505 indicates that the SH2 domain of Lck is bound intramolecularly to the phosphorylated Y505 residue.

Recall our earlier discussion of the hierarchical substructure of Lck (Figure 1). A BNGL-compliant molecular entity graph representation of Lck is shown in Figure 2A. This graph, which is drawn according to the conventions of Faeder et al. [54], includes the SH2 and SH3 domains of Lck and three tyrosine residues that can each be either phosphorylated (P) or unphosphorylated (U): Y192, Y394 and Y505. As discussed previously, phosphorylation of these residues regulates the binding and catalytic properties of the protein. Note that the PTK domain of Lck is not included in this graph. The reason is that, although enzyme-catalyzed reactions can be represented in BNGL-encoded rules, explicit representation of catalytic domains is often dispensable for model specification and simulation. As a result, proteins are often represented without their catalytic domains for simplicity, as shown in Figure 2A. Briefly, other features of Figure 2A are as follows. Nodes are colored: they share the color "Lck." To avoid actual use of color, the nodes are surrounded by a box. Tildes proceed the possible states of a component; here, tyrosine residues may be phosphorylated (P) or unphosphorylated (U).

In Figure 2B, a hierarchical graph representation of Lck that corresponds to Figure 2A is shown. The directed edges in Figure 2B represent containing or ownership relations. In Figure 2B, the PTK domain of Lck is explicitly represented, so that membership of Y394 in the PTK domain of Lck is clear. Similarly, one can see that Y192 is part of the SH2 domain of Lck. In this graph, possible internal states are indicated in boxes attached to the bottoms of component boxes, which is consistent with the conventions of Hu et al. [40].

A chemical species graph is a complete specification of a molecule or a molecular complex, including internal states. Figure 2C shows a chemical species graph for free Lck in which Y192 and Y394 are unphosphorylated (U) and Y505 is phosphorylated (P). The hierarchical graph representing this chemical species suggests to a reader that intramolecular binding between the SH2 domain and phosphorylated Y505 may affect the kinase activity of Lck, because the kinase domain is located between the SH2 domain and Y505 in the layout of the graph, which is consistent with ordering of components from the N-to the C-terminus of the polypeptide chain.

Both of the hierarchical representations shown in Figure 2 (panels B and C) capture the essential information of the protein sequence illustrated in Figure 1A: the internal relationships among the domains and residues of Lck. It differs from a non-hierarchical BNGL-encoded representation of the molecule, such as LCK(SH3, SH2, PTK, Y192, Y394, Y505), which tells us nothing about how the tyrosine residues relate to the domains. In contrast, in the hierarchical representation, one can see that Y192 is inside the SH2 domain. One can also see that Y505 is a tyrosine residue located at the C-terminus of the kinase domain, although this feature derives from the layout of the graph.

#### Hierarchical graph representation of the TCR complex

**Figure 3:**
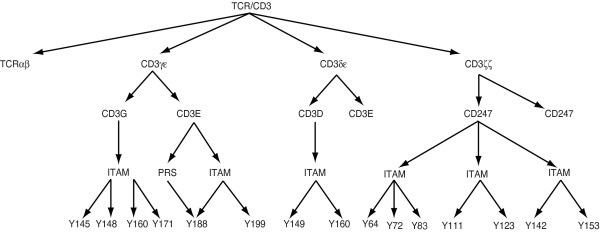
**A hierarchical graph representation of the TCR complex**. This hierarchical graph represents the TCR complex. Each of the dimeric subunits (TCR*αβ*, γϵ, *δ*ϵ, ζζ) is represented as a subgraph. The name of the dimeric subunit is shown in the root node of the corresponding subgraph. Arrows indicate containing relationships. Tyrosine 188 (Y188) belongs to two different linear motifs (the PRS and ITAM of the CD3ϵ chain). Note that only one polypeptide chain is drawn beyond the component molecules level to avoid redundancy.

To represent a multimeric protein like the TCR complex, we can represent each of its constituent polypeptide chains as a hierarchical graph, as demonstrated above for Lck. The hierarchical graphs for the individual polypeptide chains can then be assembled into a larger hierarchical graph of the complex, as demonstrated in Figure 3. The root node of this graph indicates that the name of this molecular complex is TCR. Nodes in the next layer show the names of the constituent subunits, which are homodimers and heterodimers. In the third layer, each node represents a single polypeptide chain that is part of a dimer in the second layer. The fourth layer lists the linear motifs in those polypeptides and the fifth layer lists amino acid residues that belong to the linear motifs in the fourth layer. Thus, complexes can be represented by hierarchical graphs. From this hierarchical graph it is obvious that Y188 appears in both the PRS and ITAM of CD3ϵ. Thus, it can be inferred that interactions involving Y188, the ITAM, and the PRS may regulate one another. This is in fact the case, as discussed earlier.

### Algorithm for canonically labeling hierarchical graphs

**Figure 4:**
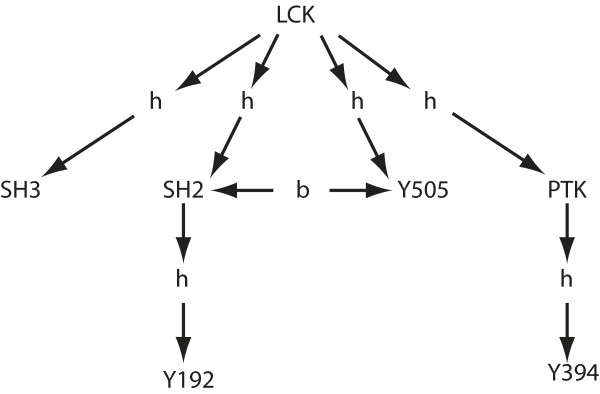
**Conversion of a hierarchical graph to a regular flat graph**. A method of reducing a graph with many edge types to a graph (on more vertices) with only one edge type. Compare with Figure 2C. In this figure, a "b" vertex corresponds to a bond, and an "h" vertex corresponds to a structural relationship.

Above, we proposed that models of signal transduction networks should make use of graphs with two types of edges; one expressing the structural hierarchy of molecular components, the other the (non-covalent) bonds between components. Thus, the edges of these graphs will be labeled either "hierarchy" or "bond." It is important to be able to use hierarchical graphs not just for improved annotation but also to incorporate them into executable models in the future. There are two methods to incorporate hierarchical graphs (and in general graphs with multiple edge types) into a computational setting. The first is to "flatten" the graph by removing the labels of all the edges, so that there is only one edge type. This simplification can be accomplished without losing the information contained in the edge labels. For each edge, we can insert a new vertex into the graph, labeled to indicate that edge's type. In particular, for an edge *e *of type *l *connecting the vertices *x *and *y*, we can delete e from the graph and insert a new vertex *v*. We can give *v *the label *l *and connect it to both *x *and *y*. Performing this step for every edge in the original graph produces a bipartite graph with one edge type. Although no information is lost using this method, it is visually inelegant and obscures the model (cf. Figure 2C and Figure 4).

Another method which preserves the clarity of the original model is to simply keep the original edge types. To make the model executable, we can assign distinct values to each edge type from 0 to *m*, where *m *is the number of distinct edge types. Then the graph can be represented by the following sort of adjacency matrix. Set the *ij^th ^*entry of the adjacency matrix to be ∑2*^e ^*taken over edges with type e between vertices *i *and *j*. Note that a non-directional edge e between vertices *i *and *j *will contribute to both the *ij^th ^*entry and the *ji^th ^*entry, whereas a directed edge e from *i *to *j *(i.e., a hierarchy edge) will only contribute to the *ij^th ^*entry of the adjacency matrix. A graph can be reconstructed from such an adjacency matrix under the assumption that the graph does not contain multiple edges (more than one edge between a given pair of vertices) of the same edge type. For biological models, the restriction to such graphs is natural. Because our main motivation for using hierarchical graphs to model biochemical networks is to improve the clarity of models, we recommend this second method.

For instance, consider the chemical species graph of Lck with SH2 connected to phosphorylated Y505 (Figure 2C). Let the hierarchy edges be edge type 0 and the bond edges be edge type 1. Then in the adjacency matrix, a hierarchical edge from *i *to *j *will be represented by a 1 = 2^0 ^in the *ij^th ^*entry, whereas a bond edge between vertices *i *and *j *will be represented by a 2 = 2^1 ^in both the *ij^th ^*and the *ji^th ^*entries. The adjacency matrix (for the species graph of Lck shown in Figure 2C) is given in Table 1.

**Table 1:** Adjacency matrix for the chemical species graph of Figure 2C.

	Component						
	
Component	Lck	SH3	SH2	Y505	PTK	Y192	Y394
Lck	0	1	1	1	1	0	0
SH3	0	0	0	0	0	0	0
SH2	0	0	0	2	0	1	0
Y505	0	0	2	0	0	0	0
PTK	0	0	0	0	0	0	1
Y192	0	0	0	0	0	0	0
Y394	0	0	0	0	0	0	0

The numerical entries in this table comprise a 7 × 7 matrix.

As can be seen from the first row of the matrix in Table 1, Lck contains SH3, SH2, Y505 and PTK components. Likewise, from the third and fourth rows, one can see that the SH2 component contains a Y192 subcomponent and the PTK component contains a Y394 subcomponent. From the pair of "2" entries, one can see that there is a bond between the SH2 and Y505 components.

An important capability of BioNetGen is the ability to distinguish between different graphs and to recognize isomorphic graphs [51]. We describe a slight generalization of the Nauty algorithm which can canonically label graphs with several edge types. This algorithm, HNauty, has been incorporated into BioNetGen [29]. Although our algorithm is only slightly different from the one described by McKay [55], we provide a brief description of the whole algorithm for clarity.

Although the representation of graphs within computing systems can vary, it is useful to think of a graph as being represented by an adjacency matrix for the graph. However, the same graph can have several different adjacency matrices associated with it; different permutations (i.e., orderings) of the vertices may correspond to different adjacency matrices. If a graph is represented by an adjacency matrix, the problem of finding a canonical label for a graph is thus nothing more than picking a canonical adjacency matrix for each graph, that is a canonical permutation of the vertices. This can be done by brute force; there are *n*! permutations of the vertices of a graph with n vertices. Each permutation corresponds to a possibly unique adjacency matrix. The adjacency matrices can be linearly ordered by considering each matrix as a binary string of length *n*^2^. The first (smallest) such string can then be chosen as the canonical label (matrix representation) for the given graph. The problem with this method is that it involves producing and sorting *n*! strings.

#### Comparing Graphs: Graph Isomorphism and Graph Automorphism

**Figure 5:**
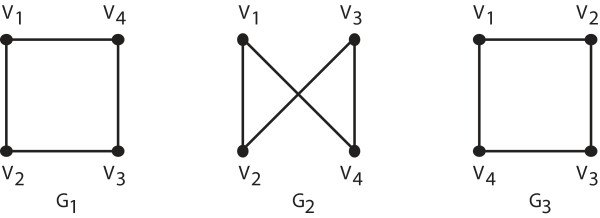
**Three isomorphic graphs**. The permutation producing the third graph from the first is an automorphism of the first graph. However, this is not true of the permutation producing the second graph from the first graph.

Graph isomorphism is closely related to graph automorphism; both play an important role in the Nauty algorithm. We will define and briefly explain both concepts. Two graphs *G *and *H *are isomorphic if there is a bijection *ϕ *(a one-to-one and onto map) between the vertex sets of the two graphs such that (*u*, *v*) is an edge of G if and only if (*ϕ *(*u*), *ϕ *(*v*)) is an edge of *H*. It is convenient to associate each isomorphism of a graph *G *to a permutation of the vertices of *G*; a graph with *n *vertices has *n*! isomorphic copies. A permutation π, of the vertices, is an automorphism of the graph *G *if each pair of vertices (*u*, *v*) is an edge of G if and only if the pair (π(*u*), π(*v*)) is also an edge of G. The automorphisms of the graph are a formal description of the symmetries of the graph. For instance, two vertices are symmetric in the graph if there exists an automorphism which maps one vertex to the other. These concepts are illustrated in Figure 5. The second and third graphs are isomorphic copies of the first. However, the second is not generated by an automorphism of the first whereas the third is.

Let us exam the graphs in Figure 5 more carefully. The permutation, π_1_, generating *G*_2 _from *G*_1 _is defined as follows: π_1_(*v*_1_) = *v*_1_, π_1_(*v*_2_) = *v*_2_, π_1_(*v*_3_) = *v*_4 _and π_1_(*v*_4_) = *v*_3_. The permutation, π_2_, generating *G*_3 _from *G*_1 _is defined as follows: π_2_(*v*_1_) = *v*_1_, π_2_(*v*_2_) = *v*_4_, π_2_(*v*_3_) = *v*_3 _and π_2_(*v*_4_) = *v*_2_. Note that π_2 _satisfies the definition of an automorphism of the graph *G*_1 _whereas π_1 _does not. The set of those permutations of the vertices that are automorphisms of a graph *G *is called the automorphism group of the graph *G*. The size of the automorphism group of a graph is a measure of the amount of symmetry in the graph. As will become clear later, it is often more difficult to find canonical labels for graphs that are highly symmetric (i.e., graphs that have large automorphism groups) than for graphs with small automorphism groups.

#### Determining graph isomorphism

A common element of methods for both assigning canonical labels and determining graph isomorphism is the individualization and refinement procedure [55,65]. The Nauty algorithm also uses this procedure which is outlined below. The procedure involves partitions; a partition *P *of a set *S*, is a collection of non-empty pairwise disjoint subsets {*S_i_*} of *S *whose union is *S*. The sets *S_i _*are referred to as the cells of the partition. An ordered partition is formed by ordering the cells of a partition. In other words, the ordered *k*-tuple (*S*_1_, *S*_2_,..., *S_k_*) is an ordered partition of *S *if the sets *S_i _*form a partition of *S*. Unless otherwise specified, in what follows, we will always use "partition" to refer to an ordered partition. The length of a partition is the number of cells in the partition. A partition is discrete if its length is equal to the size of the set *S *and trivial if it has only a single cell. A discrete partition of the vertex set of a graph is an ordering of the vertices and thus equivalent to a permutation of the vertex set. As the canonical labeling problem is equivalent to finding a canonical permutation of the vertices of a graph, it is also equivalent to finding a canonical discrete partition of the vertex set.

The individualization and refinement procedure relies heavily on so-called equitable partitions. An equitable partition is a partition *P *= (*S*_1_, *S*_2_,..., *S_k_*) of the vertices of a graph *G *such that:

(1) $$\forall i,j\in \left\{1,...,k\right\}\;x,y\in S_{i}\Rightarrow d\left(x,S_{j}\right)=d\left(y,S_{j}\right)$$

where *d*(*x*, *S_j_*) is the number of edges connecting the vertex *x *to elements of the cell *S_j _*. Similarly, *d*(*y*, *S_j_*) is the number of edges connecting the vertex *y *to elements of the cell *S_j_*. Thus, if every vertex in a graph has fixed degree k, then the trivial partition of the vertex set *P *= (V) is equitable. Note also that for any graph, every discrete partition is equitable.

There is a natural partial order on the collection of partitions of a given set *S*. Given two partitions *P *and *Q *of the same set, we say *P *is finer than *Q*, *Q *is coarser than *P*, and *P *is a refinement of *Q *(*P *≤ *Q*) if each *P_i _*is a subset of some *Q_j_*. In addition, for ordered partitions, we require *P*_1 _⊆ *Q*_1_, and ∀*i *: *P_i _*⊆ *Q_j _*⇒ *P_i_*+1 ⊆ *Q_k _*where k is either *j *or *j *+1. For example, the partition ({1, 5}, {2, 3}, {4}) is a refinement of the partition ({1, 5}, {2, 3, 4}).

As every discrete partition of a vertex set is equitable, it can be shown that every partition *P *of a vertex set has a unique coarsest refinement that is equitable [55]. This fact underpins the individualization and refinement approach. We refer to the unique coarsest refinements as equitable refinements. Consider the equitable refinements of the trivial partitions of the vertex sets of two graphs, written as *P *= (*P*_1_, *P*_2_,..., *P_j_*) and *Q *= (*Q*_1_, *Q*_2_,..., *Q_k_*). If the graphs are isomorphic, these partitions will have the same shape, meaning that for each *i*, |*P_i_*| = |*Q_i_*|. (|*S*| refers to the size of the set *S*.)

**Figure 6:**
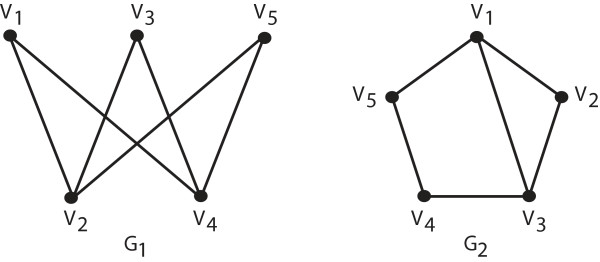
**Two non-isomorphic graphs each with five vertices**. Note that both graphs have two vertices of degree 3 and three vertices of degree 2. To see that the two graphs are not isomorphic, consider the following: we say a graph is bipartite if the vertices of the graph can be partitioned into two sets such that no edges of the graph have end points within the same partition class. For instance, *G*_1 _is bipartite; consider the partition of its vertices into the sets {*v*_1_, *v*_3_, *v*_5_} and {*v*_2_, *v*_4_}. On the other hand, it is impossible to partition the vertices of *G*_2 _into two such sets. As the property of being bipartite is invariant under permutations of the vertices of a graph, it follows that *G*_1 _and *G*_2 _are not isomorphic.

For example, let *G*_1 _be a graph with five vertices: *v*_1_,..., *v*_5 _with edges between *v_i _*and *v_j _*if *i *- *j *≡ 1 modulo 2. Let *G*_2 _also be a graph with vertices *v*_1 _..., *v*_5 _but with the edges {*v_i_, v_i_*+1} (taken modulo 5) so that we get a 5 cycle, together with an edge connecting *v*_1 _and *v*_3_. See Figure 6. Both graphs consist of five vertices; two of which have degree 3 and three of which have degree 2. (The degree of a vertex is the number of edges in the graph incident with the vertex.) Thus, by only looking at the degrees of the vertices of these two graphs, we cannot distinguish them. On the other hand, the graphs can be distinguished by finding the equitable partition of the vertex set for each graph. The unique coarsest equitable partition for *G*_1 _is ({*v*_2_, *v*_4_}, {*v*_1_, *v*_3_, *v*_5_}). Each vertex in the first cell is connected to three vertices in the second cell, and none in the first while each vertex in the second cell is connected to two vertices in the first cell and none in the second. On the other hand, the unique coarsest equitable partition for *G*_2 _is ({*v*_1_, *v*_3_}, {*v*_2_}, {*v*_4_, *v*_5_}). Here, each vertex in the first cell is connected to exactly one vertex from each of the three cells. The vertex in the second cell is connected to two from the first cell and zero from the third. As these two equitable partitions have different shapes, G1 and G2 cannot be isomorphic.

In general, equitable partitions are insufficient to distinguish between non-isomorphic graphs and therefore insufficient to determine canonical labels for graphs. They must be used together with individualization, which can be described as follows. Suppose the partition *P *is not discrete; then let *C *be the first cell of *P *with more than one element. Pick an element *x *in *C *and consider the partition *P' *formed by replacing the cell *C *with the two cells *C*\{*x*} and {*x*}. *P' *is a refinement of *P*, but it is not necessarily equitable. Thus, it is necessary to find the equitable refinement of *P'*. Continuing in this manner, it is possible to individualize and find further equitable refinements until a discrete partition is reached. As the individualized vertices were chosen at random, the procedure must be repeated for each possible choice of vertices. In this way, several discrete partitions are produced; this is the individualization and refinement procedure used in many canonical labeling algorithms including Nauty. To finish, the algorithm must select a canonical discrete partition from among those produced by the individualization and refinement procedure.

If a graph has a small automorphism group then the individualization and refinement procedure will produce only a few discrete partitions; in this case it will be relatively easy to select a canonical label. Conversely, if the automorphism group is large, the procedure will produce many discrete partitions, and it will take more effort to select a canonical label. For example, if a graph is completely symmetric then each permutation of the vertices gives an automorphism of the graph. In this case, every partition of the graph is equitable and the individualization and refinement procedure will produce each of the *n*! possible discrete partitions of the vertex set. Recall the graphs *G*_1 _and *G*_2 _considered above. The automorphism group of *G*_2 _has size 2 whereas the automorphism group of G1 has size 6. Thus, the individualization and refinement procedure produces the following two discrete partitions for *G*_2_: ({*v*_1_}, {*v*_3_}, {*v*_2_}, {*v*_4_}, {*v*_5_}) and ({*v*_3_}, {*v*_1_}, {*v*_2_}, {*v*_5_}, {*v*_4_}). On the other hand, the six discrete partitions produced for *G*_1 _correspond to those permutations of the vertices where both *v*_2_ and *v*_4_ come before the three other vertices *v*_1_, *v*_3_, and *v*_5_.

At this point it is common to use a brute-force method for finding a canonical partition from among those generated by the individualization and refinement procedure. Each generated partition *P *of the vertices corresponds to a permutation π of the vertices. Applying this permutation to the vertices of the graph, we get a new adjacency matrix A for the graph. If there are n vertices in the graph, then *A *is an *n *× *n *array of 0's and 1's. In fact, A can be considered to be a binary string of length *n*^2^. Comparing these strings as binary numbers, the smallest is selected and the corresponding partition is ordained the canonical label.

In general, the individualization and refinement procedure produces significantly less than *n*! partitions to be compared as binary strings. This efficiency is achieved because most graphs have small automorphism groups [66]. However, the method fails to significantly reduce the number of partitions that must be compared if the graph has a large automorphism group. For instance, a graph with n vertices containing every possible edge connecting these vertices has a full automorphism group, meaning that every permutation of the vertices is an automorphism. For this graph, and similarly for a graph containing no edges, the individualization and refinement procedure will completely fail to reduce the number of partitions to be compared; every discrete ordered partition will be generated by the procedure.

#### The Nauty algorithm

For highly symmetric graphs, the Nauty algorithm [55] implements a fairly effective strategy to speed up the time taken to find a canonical label. It makes use of the automorphisms of a graph to further reduce the number of partitions produced by the individualization and refinement procedure. We will now give a brief overview of the search tree used in Nauty to explain how Nauty takes advantage of knowledge of automorphisms of a graph.

Nauty takes as input a colored (labeled) graph; the coloring is taken to define a starting partition of the vertices. Nauty then builds a rooted search tree by computing successive equitable refinements of the initial partition given by the coloring. Elements of the search tree are called nodes so as not to confuse them with the vertices of the graph. The root of the search tree is the equitable refinement of the initial (given) coloring. Branches are formed by individualizing vertices and finding successive equitable refinements after each individualization step. Each movement down the search tree corresponds to individualizing an appropriate vertex and finding the equitable refinement of the resulting partition. Thus, each node at distance *k *from the root of the search tree can be represented by an ordered *k*-tuple of vertices, with the ordering corresponding to the order of vertex individualization. The leaves of the search tree (also called terminal nodes) correspond to discrete partitions. Thus, each terminal node has a natural association with a permutation of the vertices of the graph.

The key idea is that automorphisms of the graph correspond to similar leaves in the search tree. To be more precise, we say that two permutations, π_1 _and π_2_, of the vertices of the graph are equivalent if there is an automorphism of the graph, γ such that π_1 _= π_2 _* γ (that is, π_1 _is equal to the composition of the two permutations π_2 _and γ.) Then as γ is a permutation of the vertices, it can also be considered a permutation of the nodes of the search tree. (This is because each terminal node corresponds to an ordered tuple of vertices.) It can be shown that if ν is a node of the search tree, then ν^γ ^(the permutation of the *k*-tuple *ν *by γ) will be as well. In fact, much more is true: the two sets of leaves of the search tree derived from the two nodes *ν *and ν^γ ^, respectively, will be equivalent to each other. In other words, the two sets of respective permutations will be equivalent [55]. Thus, if γ is an automorphism of the graph, it is enough to produce all the terminal nodes stemming from a given node ν in the search tree, and we can ignore the terminal nodes stemming from ν^γ^. In this way, knowledge of automorphisms can be used to eliminate the need to examine (large) parts of the search tree.

Nauty discovers automorphisms in the following way. The algorithm is based on depth first search; it immediately starts generating terminal nodes. Upon producing a terminal node, Nauty applies the corresponding permutation to the original graph and then calculates the resulting adjacency matrix. Two adjacency matrices produced in this way are equal if and only if the corresponding two permutations, π_1 _and π_2_, are equivalent. In this case, there exists an automorphism γ of the graph such that π_1 _= π_2 _* γ. The Nauty algorithm then calculates γ by evaluating $\pi_{2}^{-1}*\pi_{1}$. As such automorphisms are discovered, Nauty can prune the size of the search tree as detailed above.

Nauty also uses an indicator function to further prune the search tree. An indicator function is a map defined on the nodes of the search tree that is invariant under automorphisms of the graph. This function maps the nodes into a linearly ordered set (a set in which the elements have a linear ordering, for example the real numbers.) Then Nauty skips over nodes of the search tree where the indicator function is not minimal. As the indicator function is invariant under automorphisms of the graph, a canonical label will be found among those terminal nodes of minimal indicator function value.

#### HNauty

Here we describe HNauty and explain how HNauty differs from McKay's description of Nauty [55]. One difference is that, as HNauty allows for several different edge types, the adjacency matrices associated with the graphs in HNauty may contain not only 0's and 1's for entries but can have entries of the form ∑2*^i ^*where *i *is taken over those edges of type *i *between the two given vertices. For a graph with two edge types (e.g., hierarchy and bond edges), the entries in the adjacency matrix can be 0, 1, 2^2-1 ^= 2^1 ^= 2 or 1+2 = 3. A value of "3" should be interpreted to mean that there is both a hierarchy edge and a bond edge between two vertices.

Another difference lies in how equitable partitions are calculated. We define a slight generalization to deal with labeled edges. A generalized equitable partition is an ordered partition *P *= (*V*_1_, *V*_2_,..., *V_k_*) of the vertices of a labeled multi-graph such that for any edge label *e*, the graph restricted to the edges labeled *e *(i.e., the subgraph consisting only of the edges labeled *e*), denoted $G{|}_{e}$, satisfies:

(2) $$\forall i,j\in \left\{1,...,k\right\}\;x,y\in \;V_{i}\Rightarrow d\left(x,\;V_{j}\right)=d\left(y,V_{j}\right)$$

where *d*(*x*, *V_i_*) is the number of edges labeled e between *x *and *S_i_*. In other words, the partition is equitable with respect to the graph restricted to any single edge type. It can be proved that, given a partition *P*, there exists a unique coarsest generalized equitable refinement of *P*. To see this, note that it is enough to prove it for un-ordered partitions. (All of the partitions in Nauty and HNauty are ordered, and the orderings are canonical: sets with higher degrees come before sets with lower degrees.) Now, suppose that *Q*_1 _and *Q*_2 _are both generalized equitable refinements of *P*. If *Q*_1 _and *Q*_2 _are different as un-ordered partitions, then clearly their join, *Q *(in the lattice of partitions) is also generalized and equitable. In fact, a basic property of lattices implies that *Q *is also a refinement of *P*. As *Q *is coarser than both *Q*_1 _and *Q*_2_, it follows that *P *has a unique coarsest generalized equitable refinement. This property is the only property of generalized equitable partitions that is necessary to use them in place of equitable partitions.

### Implementation

Except for using generalized equitable partitions in place of equitable partitions, our implementation follows the description given by McKay [55]. Apparently, the actual Nauty program [64] contains some efficiencies not described in [55]. Thus, our algorithm is unlikely to be as finely tuned as Nauty. For an indicator function, we use the shape of the partition together with the shapes of the parent nodes in the search tree. By shape we mean the sizes of the individual cells of the partition. The partition ({0, 1}, {6}, {5}, {2, 3, 4, 7}) has shape (2, 1, 0, 1) as it has two cells of size 1, one cell of size 2, no cells of size 3 and one cell of size 4. These tuples are lexicographically ordered. This indicator function is invariant under automorphisms of the graph as required. Indeed it is invariant under any permutation of the vertices.

We implemented our algorithm in both Perl and Python. The Perl version of HNauty is available as Additional file 1. The Python version of HNauty is available as Additional file 2. HNauty is also available at the BioNetGen website [67]. The Perl version has been incorporated into BioNetGen [29]. HNauty is turned off by default in BioNetGen. It can be turned on with the command "setOption(SpeciesLabel, HNauty);" at the beginning of a BioNetGen input file. The default for BioNetGen is to calculate pseudo-canonical labels that do not distinguish all isomorphic graphs but are much faster to generate than HNauty. Then any two graphs which share pseudo-canonical labels are checked for isomorphism using Ullmann's algorithm [68]. The generation of pseudo-canonical labels followed by applying Ullmann's algorithm to graphs with the same label always produces correct results, though it can be much slower than HNauty if a chemical species graph is composed of many isomorphic subgraphs. The HNauty code can be run as stand-alone code separate from BioNetGen. The Python version of HNauty uses the graph structures defined in the freely available package NetworkX [69]. The Perl version of HNauty takes as input the graph adjacency matrix together with an initial partition of the vertices of a graph. The adjacency matrix should be in the form of a dictionary of dictionaries. The keys of the first dictionary are the vertices of a graph. Each vertex *i *points to a second dictionary whose keys are the neighbors of vertex *i *in the graph. In this second dictionary, a vertex *j *points to an array containing the edge types between vertices *i *and *j*. (For graphs without multiple edges, each such array will only contain one edge type.) The initial partition of the vertices should be given in the form of an array of arrays, each of the smaller arrays being a set in the partition. HNauty returns as output a permutation of the vertices of the input graph. Permuting the input graph under this permutation gives the canonical label of the graph (i.e., the resulting adjacency matrix is the canonical label).

### Testing

Both the Python and Perl versions of HNauty were extensively checked using a database of isomorphic graphs [70]. The Perl version was further checked against randomly generated graphs with two types of edge: directed and undirected. These graphs were generated using the Erdős-Rényi model for random graphs; the edges were chosen independently with uniform probability. Edges were selected to be undirected with probability 0.1 and directed with probability 0.05. With probability 0.85 an edge was not in the graph. One thousand graphs, each on two hundred nodes, were produced in this way. Each was given as input to HNauty and then a random permutation of the vertices was applied to each graph; the result was also given as input to HNauty. A test was successful if the two isomorphic inputs resulted in the same canonical label. All of the tests were successful.

## Discussion

In the section above, we discussed the significance of our results as the results were presented. Thus, this section will be brief. Hierarchical graphs can be powerful visual aids in understanding complex molecular structures. For rule-based models of cell signaling systems, hierarchical graphs provide more natural representations of proteins than the regular flat graphs of BNGL or Kappa and thus promote clarity in building and annotating models. Regular flat graphs can obscure the structural properties of molecules and molecular complexes. As shown above, hierarchical graphs can be used in a formal manner to model cell signaling systems. In addition, they can be incorporated into executable models in place of regular graphs. As an example, we have developed a version of the popular Nauty code which can take as input hierarchical graphs (and indeed any graphs with multiple edge types). This is important because, as noted above, determining graph isomorphism can take a significant amount of computation time in network generation. As detailed above, HNauty differs only slightly from the main outline of Nauty given by McKay [55]. Indeed, the formalism distinguishing graphs and hierarchical graphs (and graphs with two or more edge types) is also slight. Thus, we propose that the use of hierarchical graphs may, at little cost, allow for greater clarity of rule-based models for biochemical systems.

## Conclusions

The graphs and algorithm introduced here lay the groundwork for rule-based models that are easier to understand, because molecules with complicated substructures can be more naturally represented.

## Abbreviations

BNGL: BioNetGen language; PRS: proline-rich sequence; PTK: protein tyrosine kinase.

## Authors' contributions

NWL formulated, implemented and tested the HNauty algorithm. BH and WSH conceived the idea of using hierarchical graphs for annotating rule-based models and provided examples of proteins with hierarchical substructures. WSH planned the project. NWL, BH and WSH wrote the manuscript. All authors have read and approved the final version of the manuscript.

## Supplementary Material

Additional file 1 **The HNauty source code written in Perl**. Click here for file

Additional file 2 **The HNauty source code written in Python**. Click here for file
